# Acute myocarditis related to Covid-19 infection: 2 cases report

**DOI:** 10.1016/j.amsu.2021.102431

**Published:** 2021-05-26

**Authors:** O. Kallel, I. Bourouis, R. Bougrine, B. Housni, N. El Ouafi, N. Ismaili

**Affiliations:** a Department of Cardiology, Mohammed VI University Hospital of Oujda, Faculty of Medicine and Pharmacy, Mohammed the First University of Oujda, Morocco; b Department of Anaesthesiology and Reanimation, Mohammed VI University Hospital of Oujda, Mohammed the First University of Oujda, Morocco; c Laboratory of Epidemiology, Clinical Research and Public Health, Faculty of Medicine and Pharmacy, Mohammed the First University of Oujda, Morocco

**Keywords:** Fulminant myocarditis, Acute myocarditis, COVID-19, Coronavirus infections, Case report

## Abstract

**Introduction and importance:**

Since COVID 19 was described for the first time in December 2019, we have not stopped discovering its different clinical manifestations. Despite the respiratory complication which is the most common symptomatology, multi-organ dysfunction and multiple cardiovascular complications were described such as acute myocarditis, heart failure and even arrhythmias.

**Cases presentation:**

Two patients aged 26 and 56 year-old, developed acute myocarditis related to Covid-19 infection but with different symptomatology.

**Case 1:**

Presented to the emergency room with digestive symptomatology, Covid-19 infection was confirmed by a positive chest CT scan and positive COVID-19 serology testing. Clinical, biological, radiological findings allowed making the diagnosis of a Covid-19 infection with a bacterial superinfection complicated by a fulminant myocarditis.

**Case 2:**

Presented to the emergency department with a chest pain, dyspnoea, paroxistic cough, myalgia and fever. A Covid-19 infection was confirmed. The electrocardiogram showed a diffuse ST elevation, echocardiography showed normal systolic function and the high-sensitivity cardiac troponin I level was high. Invasive coronary angiography was performed, revealing angiographically normal coronary arteries.

**Clinical discussion:**

Our 2 cases were treated differently, case 1 received antibiotherapy because of the bacterial superinfection and inotropic support for the septic and cardiogenic choc. Contrarily to case 2 who received inotropic support, immunoglobulin and corticosteroid. With a total recovery for both patients.

**Conclusion:**

This article can help in considering cardiac affection due to SARS-CoV2, even with poor respiratory symptomatology, and to insist on the importance of the cardiac evaluation for young patients with a sever Covid-19 infection.

## Highlights

• COVID- 19 (coronavirus disease 2019), caused by the SARS-CoV-2 virus (severe acute respiratory syndrome coronavirus 2), was described for the first time in Whuan, China in December 2019.
• Respiratory symptoms present the most common clinical manifestation, but also multi-organ dysfunction and multiple cardiovascular complications was described such as acute myocarditis, heart failure and even arrhythmias.
• We present in this article two patients aged 26 and 56 year-old, developed acute myocarditis related to Covid-19 infection but with different symptomatology.
• Physicians should not neglect extra respiratory symptoms which can hide a COVID-19 complicated infection, and a cardiac evaluation is necessary even for young patients with a sever Covid-19 infection.

## 1 Introduction

COVID- 19 (coronavirus disease 2019), caused by the SARS-CoV-2 virus (severe acute respiratory syndrome coronavirus 2), was described for the first time in Whuan, China in December 2019, and in less than 4 months, this infectious disease was declared as worldwide pandemic, with a mortality rate estimated at 2.17% until now [1]. The first described, and most Common symptoms of this infection are fever, cough, myalgia, and/or fatigue. (2), by time, we start describing more complicated symptoms as multi-organ dysfunction and multiple cardiovascular complications. In this article we describe two cases of acute myocarditis related to a COVID-19 infection presented with different symptomatologies.

Cardiovascular involvement related to COVID-19 is less recognized and described by physicians, which can explain fears that cardiovascular complications are being underdiagnosed. This article can help in considering cardiac affection due to SARS-CoV2, even with poor respiratory symptomatology, and to insist on the importance of the cardiac evaluation for young patients with a sever Covid-19 infection.

This article has been reported in line with the SCARE criteria [3].

## 2 Cases presentation

Case 1 A 26 year-old male, with no medical, surgical or family history, nor drug history. Presented to the emergency department of Mohammed VI hospital of Oujda, Morocco, with symptoms of diarrhea, vomiting, fever, fatigue and weakness. This symtomatology started 8 days before his consultation, and was treated initially by a symptomatic treatment with no clinical amelioration.

The physical examination in the emergency room, found fever (39 °C), polypnea with 22 cycle/min of respiratory rate, SatO2 = 96% to ambient air, haemodynamic instability with a blood pressure at 80/40 mmHg, a heart rate at 120 bpm (beats per minute) and cold extremities. The rest of the cardiopulmonary examination was normal. The electrocardiogram showed a regular sinusal tachycardia at 120 bpm and negative T wave in V5–V6, with no ST-segment elevation.

The patient's nasopharyngeal swab tested negative for COVID-19 by reverse transcription polymerase chain reaction (RT-PCR), but the COVID-19 antibody testing (serology testing) was positive (for both Immunoglobulin G and M).

**Fig. 1:**
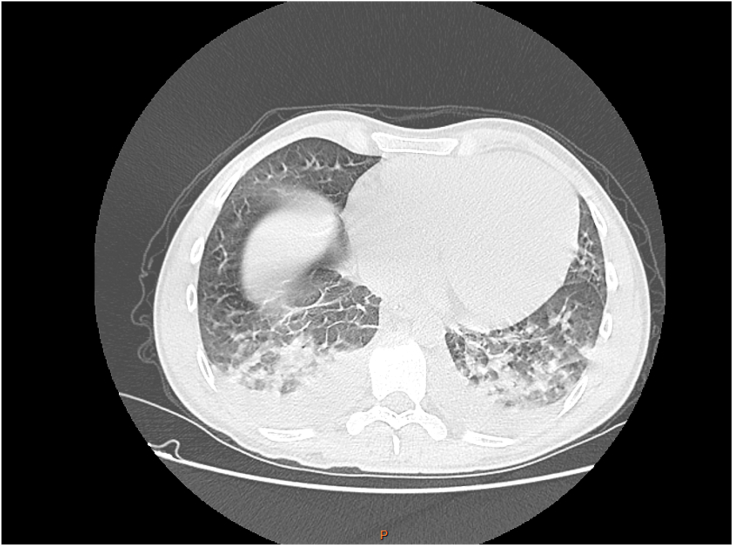
chest computed tomography showing ground-glass opacification.

The chest computed tomography showed ground-glass opacification associated to a bilateral peri-hilar condensation, a small to moderate bilateral pleural effusion and a small pericardial effusion (See Fig. 1).

All immunological tests for autoimmune diseases were negative. Other serological tests were, also, negative, including those for hepatitis B and C viruses, Human immunodeficiency Virus (HIV), Cytomegalovirus and Epstein-Barr Virus.

Markers of myocardial injury showed elevated pro BNP (brain natriuretic peptide) and troponine I. Biological assessment (See Table 1) revealed also an inflammatory syndrome, a high level of Procalcitonin and functional acute kidney failure.

**Table 1:** Biological evolution during the hospitalization.

	Case 1		Case 2		
	Initial presentation	10 days after initial presentation	Initial presentation	8 days after initial presentation	reference value
Troponine I	3867	410	677	26	26 ng/l
Pro-BNP	>25000				<125 pg/mL
C-reactive protein	500	16,8	315	32	0–5 mg/l
White cell count	14230	5800	17940	20430	4000-10000/ul
Neutrophil count	12850	3540	16370	18540	1500-7000/ul
Lymphocyte count	930	1610	560	850	1000-4000/ul
Hemoglobin	13	12,1	10,1	11,4	13–18 g/dl
Plaquet count	171000	216000	274000	253000	150000-400000/ul
Creatinine	10	10,3	45	16	7,3–11,8 mg/l
Fibrinogen	8,7	–	7,9	6.2	2–4 g/l
D-Dimers			1,04	–	<0,5 mg/l
CPK MB			19	–	0-24 UI/l
Procalcitonine	35	1.5	85	2	<2ng/mL

**Fig. 2:**
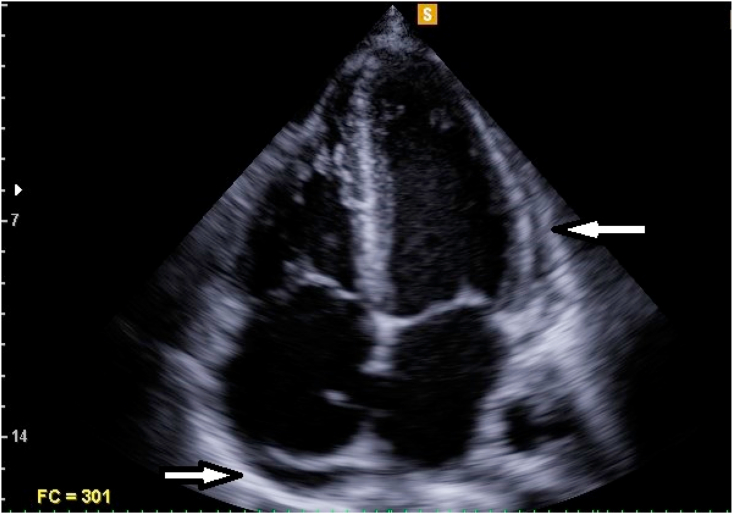
The small pericardial effusion in the first echocardiography.

The first echocardiography revealed a mildly dilated left ventricle (indexed left ventricular diastolic diameter at 32 mm/m2) with a global hypokinesia and severe systolic dysfunction: left ventricular ejection fraction (LVEF) at 30%. A dilated right ventricle with a mild systolic dysfunction, and a tricuspid regurgitation allowing calculating the systolic pulmonary pressure: SPP: 30 + 5 mmHg. With a small pericardial effusion (See Fig. 2).

The diagnosis of Covid-19 infection with a bacterial superinfection complicated by a fulminant perimyocarditis was considered (See Table 2).

**Table 2:** Summary table of diagnosis and treatment.

	Age (Years)	Covid-19 diagnosis method	Diagnosis	TTE (acute phase)	Specific treatment
Case 1	26	• nasopharyngeal swab for SARS-CoV-2 by RT-PCR: • chest computed tomography: + • COVID-19 serology testing Ig G: + Ig M: +	fulminant perimyocarditis related to Covid-19 infection with a bacterial superinfection	• Systolic dysfunction • LVEF: 30% • Dilated LV • Dilated RV with a mild systolic dysfunction. • Systolic pericardial detachment.	• Inotropic support • Antibiotherapy Then: • heart failure treatment: bisoprolol and Perindopril
Case 2	56	• nasopharyngeal swab for SARS-CoV-2 by RT-PCR: + • chest computed tomography: +	fulminant myocarditis related to the Covid-19.	• Normal systolic function • No pericardial effusion	• Inotropic support • Immunoglobulin • corticosteroid • azithromycine

In response to the hemodynamic instability due to septic and cardiogenic choc, the patient was hospitalized in the intensive care unit, and was treated by Dobutamine (5 γ/kg/min) and noradrenaline (1 mg/h), with a significant amelioration of the hemodynamic parameters in the first 48 hours, associated to a graduated decrease of the inotropic support, that was stopped by the third day.

Since the patient didn't develop any remarkable desaturation, only oxygen therapy was indicated, with no need for invasive ventilation.

Antibiotherapy was quickly started associating ceftriaxon (during 7 days) and ciprofloxacine (during 10 days) with a remarkable clinical amelioration of the sepsis and regression of the inflammatory markers (See Table 1).

The control trans-throracic echocardiography (TTE), in the 5th day of the hospitalization, showed a significant amelioration of the LVEF (45%), the LV was no more dilated with a normal wall motion, and no pericardial effusion. No electric modification in the ECG.

The patient was discharged, after 10 days of hospital stay, with heart failure treatment associating bisoprolol 5mg/day and perindopril 5 mg/day.

The Cardiac magnetic resonance imaging (MRI) was not available during the acute phase, and it was realised until 7 weeks after the discharge and it was normal.

Three months after his discharge a control TTE showed an amelioration of the LVEF to 55%, the LV and the RV were not dilated, with a normal wall motion.

Our patient was satisfied with the quality of our management.

Case 2 A 56 year-old man was referred to our Mohammed VI hospital of Oujda, Morocoo from a periphery hospital, for a chest pain, dyspnoea, paroxistic cough, myalgia, fever and severe asthenia. The patient had history of diabetes (for 10 years), obesity (BMI: 30 kg/m2) and abdominal obesity, but no drug nor psychosocial or family history.

The physical examination, on arrival at the emergency room, find fever measured at 38.7°, Glascow score at 12/15, polypnea with 25 cycle/min of respiratory rate, and a SatO2 = 80% to ambient air. Haemodynamic parameters were unstable with blood pressure at 80/45 mm Hg, heart rate at 140 beats per minute (bpm).

The rest of the physical examination was unremarkable.

A chest computed tomography showed typical findings of COVID-19 with ground-glass opacification. A nasopharyngeal swab was positive for SARS-CoV-2 by RT-PCR.

The electrocardiogram showed sinus rhythm at 133 bpm, with a diffuse ST elevation and simple monomorphic supraventricular extrasystoles. Laboratory data showed elevated levels of the high-sensitivity cardiac troponin I.

TTE was normal: normal systolic function with a normal wall motion, with no pericardial effusion and invasive.

Coronary angiography was performed by a senior Cardiologist, revealing angiographically normal coronary arteries. The intervention adherence and tolerability were well.

Cardiac magnetic resonance imaging (MRI) was not available in our centre.

Clinical, biological and radiological data suggested the diagnosis of acute myocarditis related to the Covid-19 (See Table 2).

The patient was hospitalized in the intensive care unit. In response to the respiratory failure at the admission, an oxygen therapy with a high concentration mask (10 litters/minute) was quickly started, and the Haemodynamic instability required the administration of dobutamine (5 γ/kg/min) and noradrenaline (3 mg/h).

The patient was treated, also, by immunoglobulin (one dose of 80mg of Tocilizumab), corticosteroid, and azithromycine (500mg the first day then 25 mg/day for 4 days).

Inotropic support was gradually decreased, and then stopped by the third day, with a significant amelioration of the haemodynamic parameters.

After 3 days in the intensive care unit, the patient was transferred to the “stable Covid-19 unit”, and discharged, 7 days later, on anticoagulant treatment (Enoxaparin 100 UI/Kg/12h for a week after discharge), with abated symptoms and decreased level of troponin (26 ng/l).

Our patient was satisfied with the quality of our management. Moreover, no other interventions were performed.

## 3 Discussion

Myocarditis is defined as an inflammatory disease affecting the heart, characterized by an inflammatory infiltrates and myocardial injury apart from an ischemic cause. But the exact physiopathology and mechanisms of COVID-19 related myocarditis is not clearly understood. Current knowledge propose that it’s a combination between systemic inflammation with a cytokine storm, myocardial harm caused by the patient's immune response and direct viral injury of the myocardium (since the identification of the viral particles of SARS-CoV-2 in some patient's RT-PCR myocardic biopsy) which suggests the eventual cardiotropism of the virus [4].

Case 1 presented symptoms of diarrhea, vomiting, fever, fatigue and weakness, but with no difficulty in breathing or dry cough, contrary to Case 2 who presented more common symptomatology of COVID-19 infection: fever, dyspnea, cough, myalgia and asthenia. That can suggest that COVID-19 can be involved in sever cardiac injury, even in the absence of respiratory symptoms [4].

Some studies suggest that sever COVID 19 infections with cardio-vascular complications are mostly observed 8–15 days after the beginning of the symptomatology [4]. That is consistent with the Case 2, who presented digestive symptoms 8 days before his consultation. This duration of 8 days can explain, also, the negative nasopharyngeal swab test.

Jia-Hui Zeng et al. [2] reported in April 2020 one of the first cases of fulminant myocarditis due to COVID-19, it was a 63 yers-old man, who presented elevated troponin I with decreased LVEF in TTE. He was treated with antiviral therapy and mechanical life support, with a significant biological and cardioechographic amelioration, but the patient died from a secondary infection.

Ever since, other cases of acute myocarditis was reported with different degrees of severity ranging from a total recovery as of the first week after initiation of treatment (like the case reported by Inciardi et al. [5]), to more severe cases recovering in 3–4 weeks, or to death due to cardiogenic shock. (2.6.7.8).

In our both cases, we noticed increased troponin I, which is reported in almost all the cases reporting a COVID-19 related myocarditis. And some studies suggest that the increase troponin level was observed in severe cases much more than others. [9,10].

Inciardi et al. [5] Hu et al. [6], Yokoo et all [7], Kim et all [11], and the vast majority of cases presenting COVID-19 related myocarditis reported a left ventricular dysfunction on the TTE. That was reported also in Case 1, who reported also a RV systolic dysfunction.

Otherwise, in Case 2, the TTE was normal with no systolic dysfunction. Jean-François Paul et all [12] reported, also, a case of acute myocarditis due to COVID-19 with a normal systolic function in the echocardiography.

There are no evidences in the literature about a clear and unique therapeutic consensus for the COVID-19 related myocarditis. Different treatments were proposed after the first case reported of acute myocarditis caused by a COVID-19 (steroids, intravenous human immunoglobulin, antiviral therapy, inotropic support, interferon alpha-1b, methylprednisone), with almost similar results. [2,5,6,7,8].

Our 2 cases was treated differently, Case 1 received antibiotherapy because of the bacterial superinfection and inotropic support for the septic and cardiogenic choc. Contrarily to Case 2 who received inotropic support, Immunoglobulin, corticosteroid, and azithromycin. With a total recovery for both patients.

The difference of therapeutic attitudes, leading to similar results, makes us question the efficiency, necessity and the real value of some new treatments for myocarditis linked to Covid, that different centres propose.

Notably, we consider that the lack of radiological evidences using the cardiac MRI, during the acute phase of the Covid-19 infection, is one of the limitations of our two cases report.

## 4 Conclusion

Both of our cases presented an acute myocarditis related to a Covid-19 infection, but with different symptomatology. Regardless the poor respiratory symptomatology presented by Case 1, a sever cardiac complication was diagnosed. This raises the possibility of a cardiac affection due to SARS-CoV2 even with no alarming respiratory symptomatology, and the necessity of a cardiac evaluation even for young patients with a sever Covid-19 infection.

## Ethical approval

The ethical committee approval was not required give the article type (case report). but we notice that the written consent to publish the clinical data of the patients was given and is available to check by the handling editor if needed.

## Sources of funding

None.

## Author contribution

Kallel Oussama: Study concept, Data collection, Data analysis, writing the paper. Ichrak Bourouis: Data analysis. Ramia Bougrine: Data collection. Brahim Housni: data validation. El Ouafi Nouha: data validation. Ismaili Nabila: Supervision, data analysis and data validation.

## Research registration

This is a medical case report not an original research project. This registration is not required.

## Guarantor

Kallel Oussama.

## Patient consent

Written informed consent was obtained from the patient for publication of this case report and accompanying images. A copy of the written consent is available for review by the Editor-in-Chief of this journal on request.

## Provenance and peer review

Not commissioned, externally peer-reviewed.

## Declaration of competing interest

There are no conflicts.
